# Machining Characteristics During Short Hole Drilling of Titanium Alloy Ti10V2Fe3Al

**DOI:** 10.3390/ma17225569

**Published:** 2024-11-14

**Authors:** Michael Storchak

**Affiliations:** 1 Institute for Machine Tools, University of Stuttgart, Keplerstraße 7, 70174 Stuttgart, Germany; mstorchak02@gmail.com; Tel.: +49-176-485-21079; 2 Department of Mechanical Engineering, Zhytomyr Polytechnic State University, Chudnivska Str. 103, 10005 Zhytomyr, Ukraine

**Keywords:** machining, short-hole drilling, cutting force, cutting power, chip morphology, cutting temperature, finite element method, simulation

## Abstract

The single-phase titanium ß-alloy Ti10V2Fe3Al (Ti-1023) has been widely used in the aerospace industry due to its unique mechanical properties, which include high fatigue strength and fracture toughness, as well as high corrosion resistance. On the other hand, these unique properties significantly hinder the cutting processes of this material, especially those characterized by a closed machining process area, such as drilling. This paper is devoted to the study of the short hole drilling process of the above-mentioned titanium alloy using direct measurements and numerical modeling. Measurements of the cutting force components in the drilling process and determination of the resultant cutting force and total cutting power were performed. The macro- and microstructure of chips generated during drilling were analyzed, and the dependence of the chip compression ratio and the distance between neighboring segments of serrated chips on cutting speed and drill feed was determined. Experimental studies were supplemented by determining the temperature on the lateral clearance face of the drill’s outer cutting insert in dependence on the cutting modes. For the modeling of the drilling process using the finite element model, the parameters of the triad of component submodels of the numerical model were determined: the machined material model, the model of contact interaction between the tool and the machined material, and the fracture model of the machined material. The determination of these parameters was performed through the DOE sensitivity analysis. The target values for performing this analysis were the total cutting power and the distance between neighboring chip segments. The maximum deviation between the simulated and experimentally determined values of the resulting cutting force is no more than 25%. At the same time, the maximum deviation between the measured values of the temperature on the lateral clearance face of the drill’s outer cutting insert and the corresponding simulated values is 26.1%.

## 1. Introduction

The cutting processes of various materials are the most common methods of forming parts for machines and mechanisms and ensuring the properties of these parts that are necessary for their functioning [1,2]. Successful application of cutting processes relies on complete information about the physical mechanisms and patterns that occur directly in the cutting zones. The machining processes of materials by cutting manifest these mechanisms and regularities using the thermo-mechanical characteristics of these processes, which are the analyzed object of numerous studies [3]. The deepest understanding of the physical mechanisms accompanying the cutting process is provided by the symbiosis of experimental and modeling research methods. At the same time, the modeling of cutting processes and especially their numerical simulation, which has been developing rapidly recently, provide the possibility of estimating thermo-mechanical characteristics simultaneously with reducing the costs of complex experimental studies [4,5].

Significant difficulties arise when evaluating the machinability and investigating the cutting process of such difficult-to-machine materials as titanium and nickel alloys [6,7], as well as when evaluating the workability of the tools used in the machining process [8,9]. The mentioned difficulties are aggravated when determining the thermo-mechanical characteristics of the spatial cutting processes of difficult-to-machine materials, which undoubtedly include milling [10,11] and drilling [12,13] processes. The machining of new titanium alloys developed recently specifically for use in the aviation and aerospace industries causes significant challenges. Such materials also include single-phase titanium ß-alloy Ti10V2Fe3Al (Ti-1023) [14], which has higher mechanical properties than, for example, the widely used titanium alloy Ti-6Al-4V. Further significant increases in the mechanical properties of the ß-phase titanium alloy are provided by its various methods of preparation, in particular, directional solidification (see, for example, [15]). Such solidification provides the possibility of creating different, for example, oriented microstructures of the specified material. This, in turn, provides significant increases in its mechanical properties.

This study is devoted to experimental and numerical analysis of the thermo-mechanical characteristics of the cutting process and the morphological characteristics of chips formed during short hole drilling in workpieces produced using single-phase ß-titanium alloy Ti10V2Fe3Al (Ti-1023).

One of the most common and oldest processes for machining materials by cutting is the drilling process [1]. A significant portion of the materials subjected to the drilling process are difficult-to-machine materials, particularly titanium alloys [6,16]. Various types of tools are used to realize the cutting process by drilling: twist drills (see, for example, [17,18,19]), single-edge drills for deep hole drilling (see, for example, [20,21]), tools equipped with indexable cutting inserts for short-hole drilling [22,23], and others. Numerous researchers have focused their attention on both experimental and numerical methods to determine various cutting characteristics in the drilling process of different materials. The use of the latter has significantly increased with the appearance of commercial software products for creating finite element models of various cutting processes, such as Abaqus, LS-Dyna, AdvantEdge, and Deform [3,4,5]. Kinetic and thermal characteristics, the morphological characteristics of chips generated during cutting, and the physico-mechanical and qualitative characteristics of machined hole surfaces are assumed as modeled characteristics of the titanium alloy drilling process. Yang and Sun [24], as well as Parida [25], developed finite element models of titanium alloy Ti-6Al-4V drilling to simulate the kinetic characteristics of the cutting process. The modeling of cutting temperatures during the drilling of titanium alloy Ti-6Al-4V was the subject of studies by Bonnet et al. [26] and Kumar and colleagues [27]. Marusich and his colleagues studied the components of cutting force when drilling the most commonly reported titanium alloy Ti-6Al-4V using drills equipped with indexable cutting inserts [23]. In addition to the kinetic characteristics, Svensson et al. simulated cutting temperatures and chip morphology characteristics when drilling the same titanium alloy using drills with interchangeable cutting inserts [28]. The above examples constitute only a very small portion of the studies on the numerical modeling of various characteristics of the drilling process in difficult-to-machine steels and alloys. The vast majority of these publications are united by their desire to determine the numerical model parameters that provide the smallest deviation between the simulated and measured characteristics of the cutting process.

The extent to which the results of applying numerical models to simulate the characteristics of various cutting processes sufficiently reflect the real physical processes occurring in the cutting zone fully depends on the parameters of the submodels that are components of these numerical models. These components include the model of the machined material (constitutive equation), the model of contact interaction between the tool and the machined material, which is mainly considered as a friction model, and the model of machined material fracture when the tool’s cutting elements penetrate it to generate chips.

The most widely known publications provide information about the machined material models used in numerical cutting models [5,29]. The calculation algorithms of various commercial software products include 10 to 14 constitutive equations representing different models of machined material [30]. The most common and used in the vast majority of cutting process simulations is the Johnson–Cook constitutive equation [31]. This widespread use of this equation is due to its relatively straightforward structure and the small number of parameters used compared to other machined material models. These advantages have predetermined the overwhelming use of the constitutive Johnson–Cook equation in spatial numerical cutting models. Ugur developed a three-dimensional finite element model for the drilling process of titanium alloy Ti-6Al-4V with twist drills [32]. The Johnson–Cook constitutive equation was used as a model of the machined material. The parameters of this equation were borrowed from the literature sources. Zhu and colleagues proposed a three-dimensional numerical model for drilling titanium alloy Ti-6Al-4V using twist drills to determine the cutting temperature and kinetic characteristics of the machining process [33]. The parameters of the Johnson–Cook model were also borrowed from previously published studies. Many researchers have often used this kind of methodology. Among them, it is worth mentioning the studies of Ji et al. [34], Wolf et al. [35], Kang with Yao [36], Wang et al. [37], Mugilan with Alwarsamy [38], and many others in the development of 3D cutting models of titanium alloy Ti-6Al-4V, as well as other alloys and steels. It draws attention to the fact that in most studies of the numerical modeling of the spatial cutting processes of titanium alloys, the two-phase α + β titanium alloy Ti-6Al-4V is used as the machined material. It is much less common for researchers to focus on other titanium alloys. Jaiswal and his colleagues developed a numerical model of the orthogonal cutting process of single-phase ß-titanium alloy Ti-1023 [39]. They also used the Johnson–Cook constitutive equation. The parameters of this equation were adopted from previously published studies. A spatial finite element cutting model of the titanium alloy TC21, which has mechanical properties similar to those of the Ti-1023 alloy, was proposed by Lei and Pei [40], who also used the same constitutive equation. To improve the accuracy of predicting the thermo-mechanical characteristics of the cutting process using numerical models, the classical form of the Johnson–Cook constitutive equation was modified by adding additional terms. These terms had to take into account the effects of the various physical processes accompanying the cutting process. Calamaz and colleagues proposed additionally considering the strain-softening of the titanium alloy Ti-6Al-4V during orthogonal cutting [41]. This significantly improved the accuracy of predicting the kinetic characteristics of the cutting process. The study by Cheng and Outeiro proposes a term that considers the state of stress and strain rate in the constitutive equation [42]. A significant decrease in deviation values was established by comparing the simulated and measured values of cutting forces and chip sizes generated while cutting the titanium alloy Ti-6Al-4V. Zhang and colleagues compared the predictive accuracy of cutting force and chip morphology characteristics in the orthogonal cutting of the titanium alloy Ti-6Al-4V for three different numerical cutting model formulations [43]. They determined which staging best simulates a specific characteristic of the cutting process. Pan et al. proposed a modification of the constitutive equation for the behavior of the titanium alloy Ti-6Al-4V [44] and Inconel 718 [45] that takes into account the grain size of the material and phase transformation calculations. This material model provided improved accuracy in simulating the magnitude of cutting force components and residual stresses. Feng and colleagues proposed an analytical model to predict the cutting temperature during the laser milling of the titanium alloy Ti-6Al-4V [46]. The constitutive model of the machined material behavior in the machining process takes into account the microstructural changes in this material resulting from the thermal effects of both laser machining and cutting temperature. Andrade and colleagues developed an additional term to describe the material behavior during phase transformation at the recrystallization temperature [47]. This term has been added to the Johnson–Cook constitutive equation. Calamaz et al. modified the Johnson–Cook model by adding a term to the constitutive equation that accounts for the hardening of the material at large strain rates [48]. They also supplemented the machined material model with an additional term that describes temperature-dependent flow softening. Ducobu and colleagues proposed using a modified constitutive equation that provides a more accurate simulation of serrated chip morphology [49].

For a successful simulation of the cutting process, the contact interaction model between the tool and the machined material (friction model) should sufficiently describe the sliding and sticking processes of the machined material in its contact with the tool’s rake and clearance faces. It should also account for abrasive, adhesive, and diffusion processes in this contact that cause tool wear, as well as the heat flows generated by friction between the tool and the machined material [5,29,50]. At the same time, some researchers believe that, currently, no model of contact interaction between the tool and the machined material sufficiently reflects the patterns of this contact under the conditions of the cutting process [5,29]. The absence of such a model means that a reliable and accurate method for determining the parameters of the friction model under extreme deformation, speed, and thermal conditions occurring in the specified contact during the cutting process has not yet been developed [50,51,52]. The most common friction models used in numerical cutting models are as follows: Coulomb’s model (see, for example, [53,54,55]), a model of contact interaction between the rake and clearance faces of the cutting wedge with the chip and the machined workpiece surface [56,57,58], which provides for determining the friction coefficients as the ratio of tangential to normal stresses in the seizure and sliding zones of the chip with the specified surfaces of the tool’s cutting wedge; Siebel’s model, which uses a constant shear model, assuming that the friction stress is proportional to the yield stress in shear of the machined material, with the assumption that the variation in shear and normal stress along the tool rake face is insignificant and can be neglected [59]; the Usui and Shirakashi model, which includes a nonlinear dependence of the friction stress on the material’s shear flow stress, normal stress, and contact friction coefficient in the areas of plastic and elastic contact between the tool rake face and the chip [60]; and the model by Dirikolu and colleagues, which modernizes the previous friction model by accounting for the lubrication effect, as well as the transition zone from the seizure region to the sliding region [61,62]. The above models, or their modifications, are predominantly used as friction models in numerical cutting models. The influence of these friction models on the simulated characteristics of the cutting process has been analyzed in detail in many publications. In this regard, the studies of Özel and Arrazola [51,63], the work of Childs [62], the analysis by Schulze and colleagues [52], and many others should be mentioned. Zhang et al. studied the influence of contact interaction conditions between the tool and the machined material on the modeled kinetic characteristics of the cutting process [64]. Filice and colleagues analyzed the effect of the basic friction model parameters on the simulated kinetic characteristics of the orthogonal cutting process, including the length of contact between the tool rake face and the chip, the generated chip thickness, and the shear angle [65]. An analysis of the influence of different friction models on the three most common software products used to create numerical cutting models is presented in the study by Malakizadi et al. [66]. The components of cutting forces, chip thickness, and the contact length of the tool rake face with the chip were used as simulated cutting characteristics. Afrasiabi et al. proposed modifying the Coulomb friction model using a temperature-dependent friction coefficient for the contact between the tool and the machined Ti6Al4V titanium alloy [67]. The use of this friction model significantly reduced the deviation between the simulated and measured values of the cutting force components. The contact interaction between the tool and the machined TC21 titanium alloy, as a hybrid friction model, was presented in a study by Lei and Pei [40]. In this case, the regions of the specified contact interaction were divided into a seizure region and a sliding region of the secondary cutting zone.

Concerning numerical modeling, the damage of machined material during material cutting is considered to involve two sequential processes: (a) the separation of the machined material into a chip and the material body immediately in front of the tool cutting edge at the moment when it penetrates the indicated material, and (b) the generation of a chip, the separate elements of which are connected to each other in a certain way [68,69]. The connection of the mentioned chip elements is mainly determined by the machined material and its mechanical properties. The separation of these elements is explicitly pronounced during the cutting of titanium and nickel alloys, as well as other difficult-to-machine materials. In this case, a serrated chip is generated, and the elements are connected by a shear band (see, e.g., [68,69,70]). The realization of these two processes in the computational algorithm of numerical cutting models is performed by applying different fracture models of the machined material. These models use different fracture criteria [71]. The criteria for the separation of machined material into chip and machined workpiece body during the penetration of tool cutting elements are divided into geometric and physical criteria [29,72]. The geometric criterion is realized in two ways: (a) by adding a thin layer of machined material to the geometric model of the workpiece, along which this material is divided into chips and workpiece (see, for example, [73]); in this case, the finite elements of this material layer are removed when the tool cutting wedge approaches the area where these elements are located, and (b) by remeshing the workpiece geometry model when the penetration of the tool wedge into the workpiece exceeds a predetermined value (see, e.g., [74]). The physical criterion is achieved by setting a physical value, beyond which the separation of machined material into chip and workpiece is performed [29,71]. As such, physical values, including the deformation values of the machined material, stresses in this material, and energy values, are applied. Physical criteria are primarily used in the modeling of the cutting process for difficult-to-machine materials, such as nickel and titanium alloys, as well as austenitic and hardened steels. The Johnson–Cook material fracture model is often used when the strain limit of the machined material is considered as a physical fracture criterion [75]. This model is often used for cutting hard-to-machine steels and alloys (see, for example, [70,76]). Saleem et al. applied the Johnson–Cook fracture model to the processing of the Ti6Al4V titanium alloy [76]. The parameters of this fracture model were determined based on the Recht study [77]. Ye and his colleagues applied the above model to explain the mechanism of chip shape transition from continuously serrated to discontinuously segmented [70]. Buchkremer and Schoop used a fracture criterion to determine the ratio of the plastic deformation value in the machined material, which was determined based on the Johnson–Cook fracture model, to the value of local deformation of the material [78]. The critical stress value of the Cockroft and Latham model [79], as a physical criterion for the fracture of machined material, was applied in the study by Storchak and colleagues [80]. The energy criterion for modeling ductile fracture of titanium alloy was used in the study of Chen et al. [81]. The fracture energy density served as this criterion. The value of fracture energy in the cutting process of the titanium alloy Ti6Al4V was used in the study of Gamboa et al. [82]. The authors proposed an empirical equation to determine the indicated energy value. An inverse method has recently been widely used to determine the parameters of various physical criteria by comparing experimental values of selected characteristics from the cutting process with the corresponding values of simulated characteristics during the programmed enumeration of a specified set of parameters. As an example of applying the inverse method, the study of Sela et al. [83] can be highlighted. This study analyzes the application of different characteristics of the orthogonal cutting process of Ti6Al4V titanium alloy to determine the fracture model parameters of the machined material. The plastic strain value was chosen as a parameter for the fracture model.

The analysis performed indicates the necessity of determining the parameters of the triad component submodels for a particular machined material. At the same time, this analysis shows the main directions and methods of determining these parameters.

## 2. Materials and Methods

**Figure 1:**
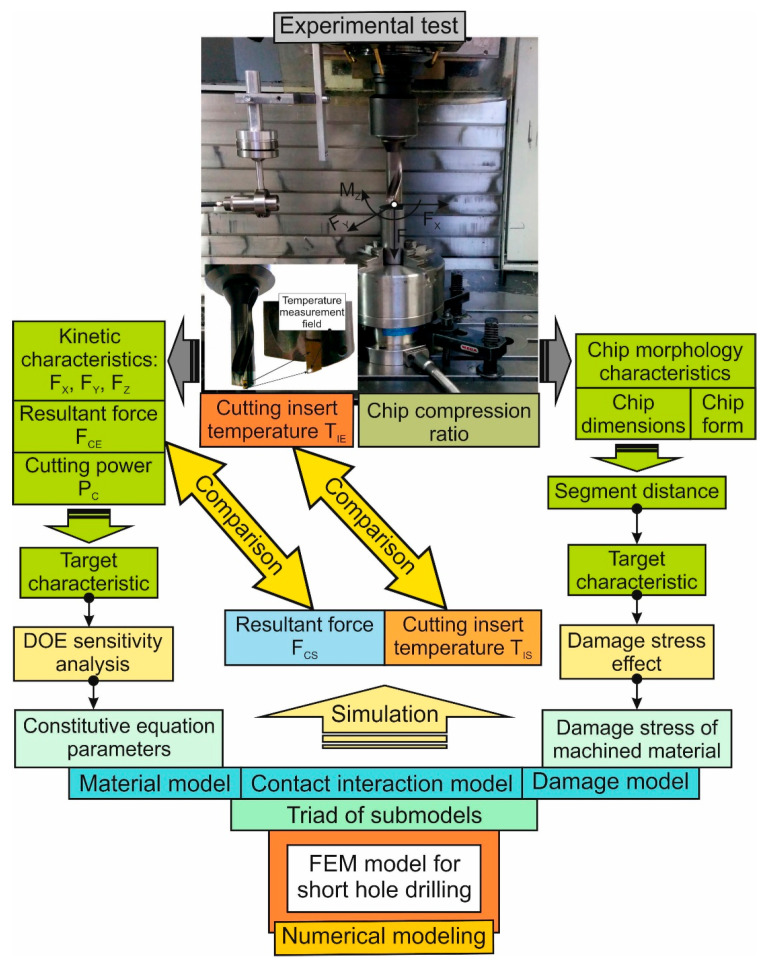
Methodology scheme for determining the thermo-mechanical characteristics of the short hole drilling process.

The drilling process of short holes in workpieces made from a single-phase (ß-phase) titanium alloy Ti10V2Fe3Al (Ti-1023) was studied experimentally and through numerical modeling using the developed finite element model of drilling [72,84]. The flowchart of the research methodology for studying the thermo-mechanical characteristics of the drilling process is presented in Figure 1. The first step of the study is devoted to experimental research. As a result of this research, the kinetic characteristics of the drilling process, such as the cutting force components *F_X_*, *F_Y_*, and *F_Z_*, were determined. The resultant cutting force *F_CE_* was determined from these components. The total cutting power *P_CE_* of the drilling process was determined from the resultant force. This power was used as the target characteristic when performing DOE sensitivity analysis [74]. At the same time, the resulting force, *F_CE_*, was used in subsequent stages of the study to determine the parameters of the constitutive equation (i.e., the parameters of the machined material model) [30]. In addition, this kinetic characteristic was used to compare the results from the cutting force measurements with the simulation of this force using an FEM model of the short hole drilling process (see Figure 1).

The first step of the study also included analyzing the characteristics of the chip morphology generated during the drilling process. These characteristics include chip dimensions, their macro- and microforms, and an integral characteristic of the cutting process—the chip compression ratio. As one of the chip size characteristics, the distance between separate segments of serrated chips generated during the machining of titanium alloys [68,69,70] was used in the subsequent stages of the study to determine the critical stress. This stress was used as a fracture model parameter for the machined material in the numerical model of drilling [71,79]. At the same step of the experimental studies, temperature measurements were performed on the lateral clearance face of the drill’s outer cutting insert. The methodology for these measurements was borrowed and described in detail in the previous study of the drilling process [84].

The second step of the study on the short hole drilling process in the titanium alloy Ti-1023 aimed to develop a numerical model for drilling. The geometric model of this numerical model and its initial and boundary conditions are mainly borrowed from previously published studies [72,84]. This step also includes determining the necessary parameters of the triad component submodels that provide an adequate simulation of the thermo-mechanical characteristics for the drilling process in the Ti-1023 titanium alloy workpieces. The machined material model (constitutive equation), friction model, and machined material fracture model are considered triad submodels (see Figure 1, numerical simulation). The parameters of the constitutive equation were determined through DOE sensitivity analysis for different values of the investigated cutting speeds and drill feeds. The generalized values of the required constitutive equation parameters for the studied range of cutting modes were determined by finding the intersection of the set values of these parameters, which were determined for each value of cutting speed and drill feed, following the methodology described in [85]. Experimental values of total cutting power *P_C_* were used as the target value when performing the DOE sensitivity analysis (see Figure 1, experimental studies). The parameters of the contact interaction model of the tool with the machined material and with the chip (friction model) [58] were determined separately for different cutting zones as coefficients of the Coulomb friction model, following the methodology described in [11]. Modeling the formation of serrated chips generated during the machining of titanium alloys [86,87] necessitates the application of the machined material fracture model [71] as one of the triad component submodels. This is predetermined by the software tool’s calculation algorithm [74] used for modeling the cutting process. The Cockroft and Latham model [79] was used as a fracture model for the machined material. The value of the critical stress, which serves as a parameter for the specified machined material fracture model, was determined through a DOE sensitivity analysis similar to the methodology outlined in [80]. The experimentally determined distance between separate chip segments was used as the target value for the DOE analysis (see Figure 1, experimental studies). The generalized value of the critical stress was also determined by finding the intersection of the value sets of the parameters determined for each magnitude of cutting speeds and drill feeds [85].

In the third step of the methodology, numerous simulations of thermo-mechanical characteristics for the short hole drilling process were performed across the entire range of the studied cutting modes. At this step, the comparison between the experimental and simulated values of the specified characteristics was also carried out, as well as the analysis of the adequacy of the developed numerical model for the short hole drilling process in the Ti-1023 titanium alloy workpieces.

### 2.1. Materials

The present study is devoted to researching the main characteristics of the cutting process, specifically the thermo-mechanical characteristics and characteristics of the chips generated during short hole drilling of high-strength titanium alloys. The representative material in this class is the titanium alloy Ti10V2Fe3Al, also known as titanium alloy Ti-1023 [80,88]. The chemical composition of this titanium alloy is provided in Table 1.

The titanium alloy Ti10V2Fe3Al, classified as a metastable β-alloy, has an ideal combination of strength, ductility, and fatigue resistance, as is typical for β-alloys. The β-stabilizing elements also give Ti10V2Fe3Al alloy good hot formability [14]. The main mechanical and thermal properties of the Ti-1023 titanium alloy are specified in Table 2. The metastable titanium alloy Ti-1023 can be forged and heat-treated at relatively low temperatures. In all experimental studies, the initial hardness of this machined material was HV 430.

Experimental studies on the short hole drilling process in the Ti-1023 titanium alloy workpieces at different cutting speeds and drill feeds were carried out at the UWF 1202 H machining center by Hermle AG, Gosheim, Germany. The experimental setup is shown in Figure 1 (experimental studies section). A detailed description of the experimental setup, the measuring system for determining the cutting force components and the temperature on the lateral clearance face of the drill’s outer cutting insert, the tool used, and the workpiece is given in previous studies [72,84]. The mechanical and thermal properties of drill cutting inserts are given in Table 2.

**Figure 2:**
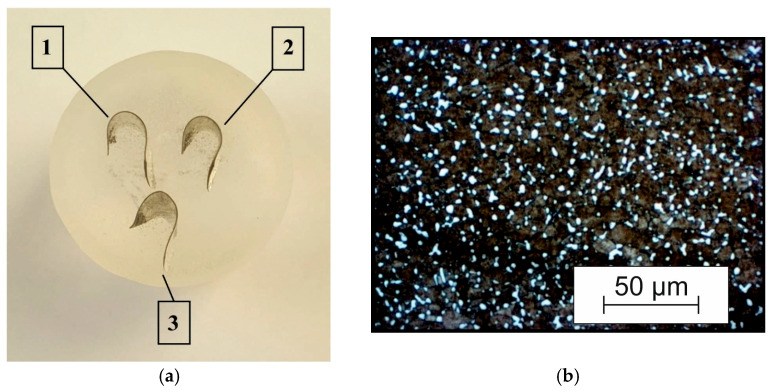
Preparatory operations for chip microstructure analysis: (**a**) slice of chips; (**b**) initial microstructure of the machined material.

Experimental studies of the drilling process were carried out by varying the nominal cutting speeds *V_C_* at four levels: 24 m/min, 48 m/min, 64 m/min, and 80 m/min. The drill feed was varied at three levels: 0.05 mm/rev, 0.10 mm/rev, and 0.15 mm/rev. The drilling process was performed without the use of coolant. Measurements of the cutting force components and the temperature of the lateral clearance face of the outer cutting insert were performed with a full factor variation in the specified cutting modes. The experimental setup described in previously published studies [72,84] was used to measure the cutting force components and the temperature of the drill’s outer cutting insert. Each test at varying cutting speeds and drill feeds was repeated at least five times. The chip generated from each test was collected for visual macroanalysis as well as microphotographic analysis of its morphology. The measurement error of the cutting force components did not exceed 11%, and the measurement error for temperature did not exceed 12%. To carry out a microphotographic analysis of the chips generated during the short hole drilling process, the chips, collected after each test, were placed in a silicone box and filled with a mixture of epoxy resin and hardener. The hardened slices were then ground and polished. Figure 2a shows a chip slice prepared using the method described above. After polishing, the slice surface was etched with a mixture of 1.5% hydrofluoric acid and 3.5% hydrochloric acid dissolved in water. The chip morphology was analyzed using a Carl Zeiss Axio Observer optical microscope from the Zeiss Group, Jena, Germany. The initial microstructure of the machined material is shown in Figure 2b.

**Figure 3:**
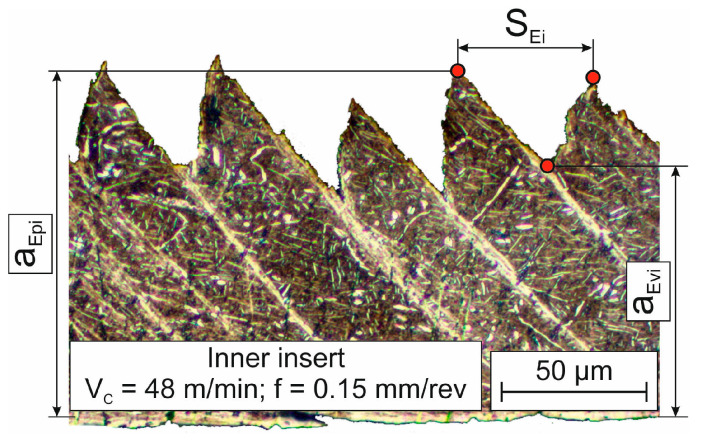
Chip microstructure example.

The chip compression ratio *K_a_* is defined as the ratio of chip thickness to drill feed. In this case, the drill feed plays the role of depth of cut in a relationship known from cutting theory [91,92,93]. Chip thickness was determined by analyzing the chip morphology from its slices. An example of the chip microstructure generated by an internal cutting insert at a cutting speed of 48 m/min and a drill feed of 0.15 mm/rev is shown in Figure 3.

The chip thickness was calculated as the arithmetic mean between the chip peaks *a_Epi_* and the chip valleys *a_Evi_*, and the distance between neighboring segments *S_Ei_* was determined according to Figure 3.

The chip compression ratio and the distance between neighboring segments used for chip characterization were determined from ten measurements of each value. The error in these measurements did not exceed 9%.

### 2.2. Methods

**Figure 4:**
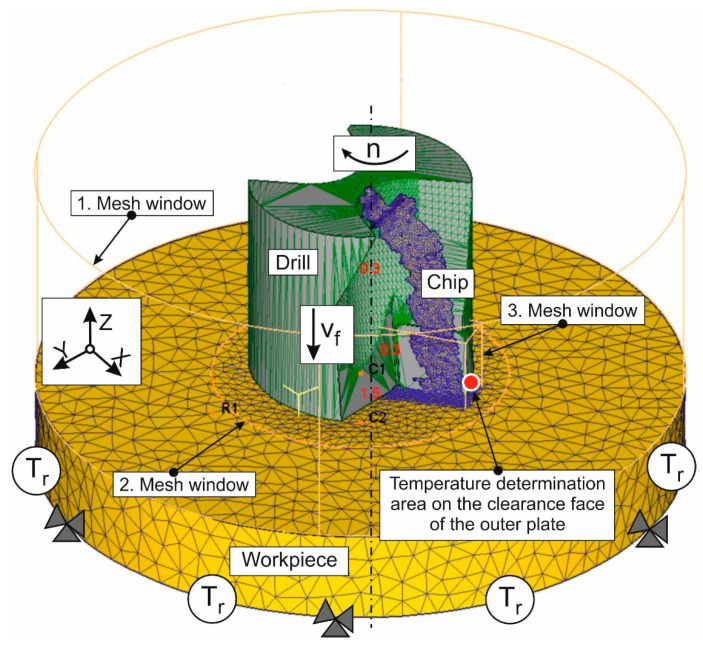
Initial geometric model of drilling process with mesh and boundary conditions, as well as with generated chip.

A finite element numerical model for the short hole drilling process was developed using an updated implicit Lagrangian formulation method. The development of this model and subsequent simulations of the drilling process were performed in the DEFORM 2D/3D™ v. 11.0 software environment [74]. The model is based on a previously developed FEM model of the drilling process, as described in [72,84]. The initial geometric model of the above numerical model and its initial and boundary conditions are shown in Figure 4. At the same time, this figure shows the chips generated during the drilling process and the mesh windows used. Additionally, Figure 4 illustrates the temperature measurement area on the outer cutting insert of the drill. The drill body and its two indexable cutting inserts were modeled as perfectly rigid bodies. At the same time, the machined material was modeled as an isotropic, plastic material. The movements of the workpiece in the developed model were constrained along all coordinate axes. The tool was given a rotary motion with a revolution frequency *n* and a translational motion in the negative direction of the Z-axis at a feed rate of *v_f_* (see Figure 4). The initial thermal conditions *T_r_* were specified along the tool and workpiece boundaries. A significantly finer mesh was used in the expected areas of the cutting zones than in the rest of the tool and the workpiece. The initial finite element model of the workpiece consisted of 152,763 elements and 29,341 nodes. The largest element side length of the workpiece model was approximately 0.789 mm. The smallest element side length of the workpiece model was approximately 0.0823 mm. The initial number of finite elements in the drill body was 36,947 and 8325 nodes. The largest element side length of the tool model was about 1.393 mm, while the smallest element side length was about 0.334 mm. The initial number of finite elements in the inserts was 23,425, with 6515 nodes. The largest element side length of the insert model was about 0.734 mm, and the smallest element side length was about 0.0842 mm. The stress–strain state of the machined titanium alloy Ti-1023 during the penetration of drill cutting inserts was described by the Johnson–Cook constitutive equation [30,31]:

(1) $$\sigma_{S}\;=\;\left(A\;+\;B\cdot \varepsilon^{n}\right)\cdot \left[1\;+\;C\cdot \ln\left(\frac{\dot{\varepsilon }}{{\dot{\varepsilon }}_{0}}\right)\right]\cdot \left[1\;-\;{\left(\frac{T\;-\;T_{0}}{T_{m}\;-\;T_{0}}\right)}^{m}\right],$$

where *σ_s_* is the yield point; *A* is the initial yield stress; *B* is the stress coefficient of strain hardening; *n* is the power coefficient of strain hardening; *C* is the strain rate coefficient; *m* is the power coefficient of thermal softening; *ε* is the actual strain; $\dot{\varepsilon }$ is the strain rate; ${\dot{\varepsilon }}_{0}$ is the reference value of strain rate; *T* is the actual temperature; *T*_0_ is the reference or room temperature, and *T_m_* is the melting temperature of the material.

DOE sensitivity analysis, embedded in the calculation algorithm of the software tool [74], was used to determine the parameters of the constitutive equation at the studied cutting speeds and drill feeds. The value of the total cutting power, determined at the corresponding values of cutting speeds and tool feeds, was used as the target value for DOE analysis. The generalized parameter values of the Johnson–Cook constitutive equation were defined as the intersection of parameter sets determined by the DOE sensitivity analysis at individual values of cutting speeds and drill feeds [85]. The contact interaction between the drill cutting inserts and the machined Ti-1023 titanium alloy was modeled using a Coulomb model [58]. The parameters of this friction model were specified using the friction windows [74,84] for the elastic and plastic regions of the secondary cutting zone and for the tertiary cutting zone. The corresponding values of these parameters (friction coefficients) were determined using the methodology presented in previously published studies [11,72]. The process of serrated chip formation, which is typical for the cutting process of titanium alloys [87], was organized by using the fracture model of the titanium alloy Ti-1023 in the calculation algorithm [71,80]. The Cockroft and Latham model [79] was used as the fracture model for the machined material:

(2) $$\sigma_{C\&L}\;=\;\int_{0}^{{\bar{\varepsilon }}_{u}}\sigma_{\max}\;d\varepsilon_{p},$$

where *σ_C&L_* is the critical fracture stress; ${\bar{\varepsilon }}_{u}$ is the ultimate plastic strain; *σ*_max_ is the maximum principal stress; *ε*_p_ is the effective plastic strain.

The critical stress at which machined material fracture occurs was determined by matching the measured and simulated values of the distance between separate chip segments at different cutting speeds and drill feeds. The generalized critical stress values were determined by the intersection of parameter sets obtained by DOE sensitivity analysis at individual values of cutting speeds and drill feeds [85]. To preserve the finite element number of the machined titanium alloy, these elements were not removed after exceeding the critical stress that corresponds to material fracture. Their carrying capacity, in this case, was taken to be equal to 10% of the initial carrying capacity of these finite elements.

## 3. Results and Discussion

**Figure 5:**
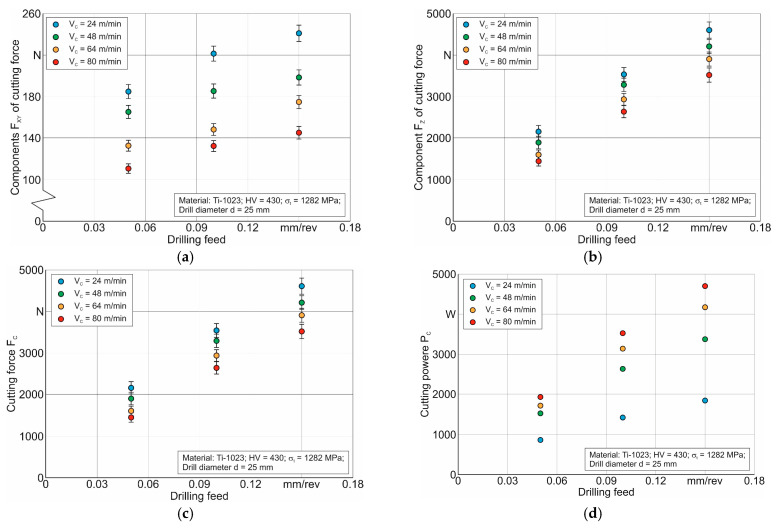
Dependence of cutting force components, their resultant, and cutting power on cutting speed and drill feed: (**a**) influence of cutting modes on the *F_XY_* component of cutting force; (**b**) influence of cutting modes on the *F_Z_* component of cutting force; (**c**) influence of cutting modes on the resultant cutting force *F_C_*; (**d**) influence of cutting modes on the cutting power *P_C_*.

Following the developed methodology for determining the thermo-mechanical characteristics of the short hole drilling process in the Ti-1023 titanium alloy (see Section 2 and Figure 1), experimental studies of cutting forces and temperature, chip morphology, and chip characteristics were performed in the first step of the study. Figure 5 shows the influence of cutting speed and drill feed on the cutting force components *F_XY_* and *F_Z_*, as well as the effect of these cutting modes on the resulting cutting force *F_C_* and on an integral characteristic of the cutting process, the total cutting power *P_C_*. From the presented diagrams, it is clear that the axial component of the cutting force *F_Z_* has a major influence on the resulting cutting force *F_C_* (see Figure 5c). Therefore, the change in the character of the resulting cutting force does not differ significantly from the change in the character of the axial component *F_Z_*. Increasing the drill feed in the studied range causes an almost proportional increase within the resulting cutting force and its components. This effect is logical because, as the drill feed increases, the amount of material removed per unit of time also increases, and therefore, the cutting work required to remove this volume of machined material also increases [91,92,93]. The resulting cutting force decreases with the increase in cutting speed in the studied range of its variation. This is most likely due to the isothermal softening of the machined material [94,95]. Such softening is caused by an increase in cutting temperature with increasing cutting speed, which is quite common in cutting processes [96,97]. The cutting power *P_C_* is an integral characteristic of the cutting process because it takes into account the combined effect of the kinetic characteristics of the cutting process and cutting speed, as well as the indirect effect of thermal machining modes [84,91]. The total drilling power *P_C_* was determined according to the following equation [92,93]:

(3) $$\;P_{C}\;=\;F_{C}\cdot V_{C},$$

Therefore, the effect of cutting speed on cutting power differs significantly from its effect on the cutting force components and their resultant. As the cutting speed increases, the cutting power increases markedly (see Figure 5d). The effect of the drill feed on cutting power is similar to that of the feed on the kinetic characteristics of the drilling process discussed above.

**Figure 6:**
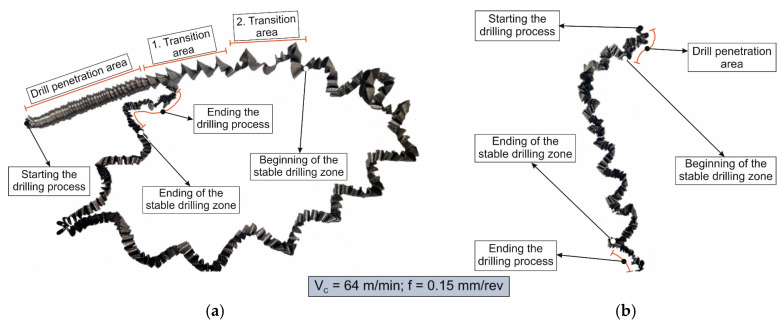
Chip shapes produced during short holes drilling in the titanium alloy Ti-1023: (**a**) chip shape formed by the outer cutting insert; (**b**) chip shape formed by the inner cutting insert.

The chip formation process during the machining of the material is responsible for generating the main thermo-mechanical characteristics of the cutting process. Accordingly, chip morphology and its fundamental characteristics have a significant impact on the successful operation of the cutting process. It is known that the short holes drilling process is carried out by two cutting inserts, usually carbide: one outer and one inner [28,98]. The cutting conditions for these inserts differ significantly from each other. Thus, the outer insert cuts the material located between the walls of the drilled hole to about half of the hole’s radius, while the inner insert removes the remaining material from the center of the hole. Thus, the average cutting speed of the outer insert is much higher than that of the inner insert. This also determines the difference in cutting temperatures, contact conditions, and other cutting characteristics between the outer and inner inserts. Different conditions of contact interaction between the drill’s outer and inner inserts and the machined material led to the generation of various chip shapes during the machining of the studied titanium alloy. Figure 6 shows the chips typically produced when drilling short holes in the Ti-1023 titanium alloy. In this particular example, the chips generated by the drill’s outer and inner cutting inserts are shown, at a cutting speed of 64 m/min and a drill feed of 0.15 mm/rev.

The chip shape changes as the drill-cutting inserts penetrate the machined material (see Figure 6). At the beginning of the drilling process, the outer cutting insert penetrates the machined material. This period is characterized by the generation of a helical chip (see Figure 6a).

As the outer cutting insert penetrates deeper into the machined material, the chip helix angle increases, characterizing the transition stage between the spiral chip shape and crimped (wavy) chips. During this period, the distance between the individual spiral elements also increases. The transitional period of chip shape change is divided into two stages: the stage of constant increase in the chip helix angle and, accordingly, the distance between the separate spiral elements, and the transition stage proper, characterized by disordered chip shaping. After the transition stage ends, a period of stable cutting, characterized by the formation of a crimped (wavy) chip, occurs. The end of the cutting process, as a result of the outer cutting insert losing contact with the machined material, is characterized by a period of the unstable cutting process due to disordered chip formation. In this case, the chip thickness decreases from the value observed in a stable cutting process to zero. The chip shape generated by the drill’s inner insert has shaping steps similar to the chip shape generated by the outer cutting insert (see Figure 6b). However, in the initial stage, a largely disordered chip structure is generated when the inner insert penetrates the machined material. Only a small section of the chips generated in this drilling step have a spiral shape. Then comes the steady shaping stage, which, similar to the chip shaping stage of the same name generated by the outer insert, is characterized by a wavy chip structure. However, in the case of chip shaping by the inner cutting insert, the pitch of the chip structure (wave pitch) is significantly smaller. The chip shape in the end-cutting region of the inner cutting insert is similar to the chip shape generated by the outer cutting insert during the final stage. It should also be pointed out that the chip length generated by the inner insert, and naturally the volume of material removed by this insert, are substantially less than the chip length generated by the outer cutting insert and the volume of material removed by this insert. This is caused by a significant difference in the average cutting speeds and, accordingly, the cutting lengths of the outer and inner inserts.

The cutting speed has a significant effect on the shape of the chip generated by both the outer and the inner cutting inserts. As a consequence of increasing cutting speed, the cutting temperature also has a significant effect [28,97]. This effect, at a drill feed of 0.1 mm/rev, is shown in Table 3. At low cutting speeds and correspondingly low cutting temperatures, spiral chip formation is observed throughout the drilling process. As the cutting speed increases, along with the corresponding rise in cutting temperature, the length of the wavy (crimped) part of the chip also increases. At the highest cutting speed of 80 m/min, the vast majority of the chips generated by the outer cutting insert are wave-shaped. At the same time, nearly the entire chip length generated by the inner cutting insert is wave-shaped (see Table 3).

A scale-up view of the effect of cutting speed on chip shape in the area of stable chip formation is provided in Table 4. The transition from a spiral chip shape to a wave-shaped chip is particularly clear for chips generated by the inner cutting insert. This is likely caused by the significantly larger gradient of speed change for the inner cutting insert.

**Figure 7:**
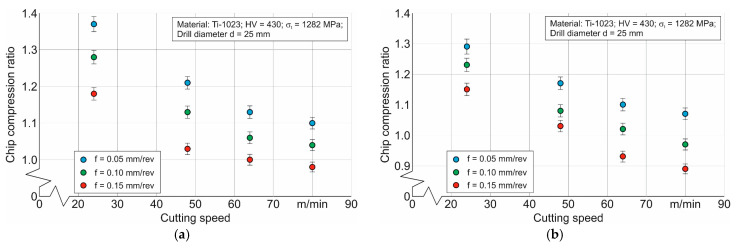
Influence of cutting speed and drill feed on chip compression ratio: (**a**) for the outer cutting insert; (**b**) for the inner cutting insert.

One of the fundamental characteristics of the cutting process is the chip compression ratio [91,92,93]. This coefficient determines many important characteristics of the cutting process, and it serves as the main defining criterion for the similarity of cutting materials [94,95]. In addition, the chip compression ratio is a criterion for the uniformity of plastic deformation during the cutting process. Determining this characteristic in spatial machining processes of titanium alloys serves as an essential addition to the analysis of the chip formation process under the specified conditions. The change in chip compression ratio with increasing cutting speed and drill feed for both the outer and inner cutting inserts of the drill is shown in Figure 7. Chip compression ratio *K_a_* decreases with increasing cutting speed and drill feed. This effect of cutting modes is observed for both the outer cutting insert and the inner insert. The observed change in chip compression ratio is due to the isothermal softening of the machined material at higher cutting speeds [94,95]. The chip compression ratio of the chip generated by the inner cutting insert is slightly less than the value *K_a_* of the chip generated by the outer cutting insert.

At high cutting speeds, chip compression ratios of less than 1.0 are observed (see Figure 7 at cutting speeds of 64 m/min and 80 m/min). In these cases, the chip thickness is less than the cutting depth (drill feed), and the chip length is greater than the cutting length; that is, the chip is not compressed but is in tension. In all probability, this is caused by the significant plastic deformation and toughness of the machined material at elevated cutting temperatures due to high cutting speeds.

**Figure 8:**
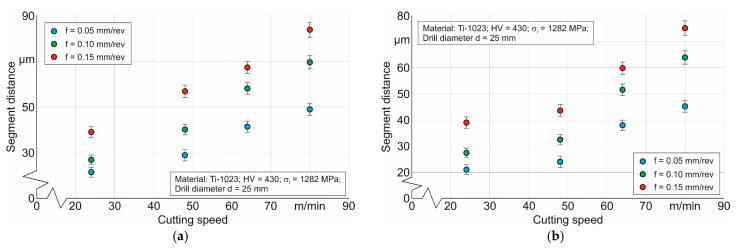
Effect of cutting speed and drill feed on the distance between neighboring segments of the generated chip: (**a**) influence of cutting modes on the distance between neighboring segments of the chip generated by the outer cutting insert; (**b**) influence of cutting modes on the distance between neighboring segments of the chip generated by the inner cutting insert.

One of the specific features of the titanium alloy cutting process is the generation of serrated chips [11,68,70] (see, for example, Figure 3), characterized by the presence of separate segments connected by a shear band. The distance between neighboring segments *S_Ei_* (see, for example, Figure 3) is an important characteristic of the generated chip that depends on the conditions of the cutting process, particularly on the cutting modes. The effects of cutting speed and drill feed on the measured characteristic *S_Ei_* values are shown in Figure 8. As cutting speed increases, the distance between neighboring chip segments also increases. This is quite logical because, as the cutting speed increases, the tool’s cutting path per unit of time also increases. At the same time, increasing the drill feed also increases the distance *S_Ei_*. In all probability, an increase in the plastic deformation of the machined material causes an increase in the formation time of the generated chip segment. It should be noted that the distance between the separate chip segments generated by the outer cutting insert is slightly greater than the corresponding distance between the chip segments generated by the inner cutting insert (see Figure 8). This is clearly explained by the difference in the average cutting speeds achieved by the outer and inner cutting inserts.

The measured values of the distances between neighboring chip segments were further used in the numerical simulation of the thermo-mechanical characteristics of the short hole drilling process to determine the critical fracture stress of the machined material as a parameter in the fracture model of this material. The measured distances *S_Ei_* were used as the target value for the DOE’s sensitivity analysis (see the description of the study methodology, Figure 1 and Section 2.2). The value of the specified target characteristic *S_Ei_* was determined as the average of the distances between adjacent segments of chips generated by the outer and inner cutting inserts.

**Figure 9:**
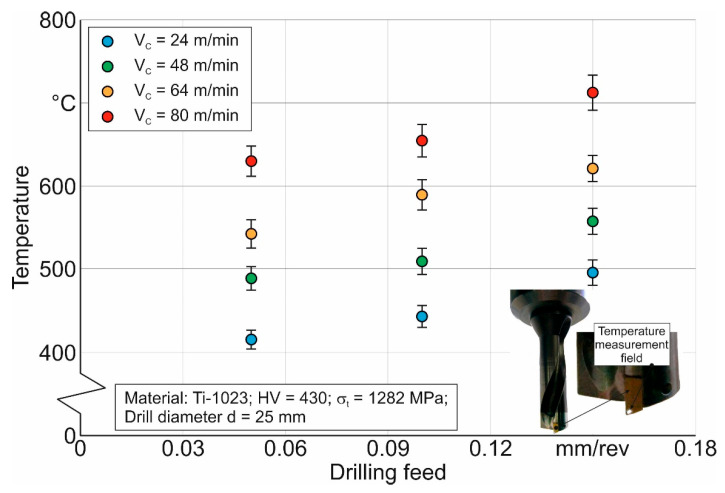
Effect of cutting modes on the temperature of the lateral clearance face of the outer cutting insert.

Following the developed methodology for determining the thermo-mechanical characteristics of the short hole drilling process in the titanium alloy Ti-1023, the final stage of the experimental studies was measuring the temperature of the lateral clearance face of the drill’s outer cutting insert [72,96,99]. The effect of cutting speed and drill feed on the outer cutting insert temperature is shown in Figure 9. Increasing the drill feed from 0.05 mm/rev to 0.15 mm/rev results in an average temperature increase of about 20%. In all likelihood, this is a consequence of an increase degree of plastic deformation in the machined material. An increase in cutting speed leads to a higher temperature acting on the lateral clearance face of the outer cutting insert during drilling. This behavior of the measured temperature change seems quite logical and is in full agreement with the established principles of cutting theory [28,91,92].

The second step of the proposed methodology for determining the thermo-mechanical characteristics of the short hole drilling process in titanium alloy (see Figure 1) is devoted to numerical modeling of the process. Numerical modeling was performed by modifying the previously developed finite element model [72,84], which was applied to the drilling process of the titanium alloy Ti-1023. The main task of modifying the finite element model of the drilling process was to determine the parameters of the triad component submodels: the material model (constitutive equation), the friction model (model of contact interaction between the tool and the machined material), and the machined material fracture model during chip formation, by dividing the machined material into chip and workpiece machined surface. The determination of the parameters for the mentioned triad components of the numerical cutting model was performed using DOE sensitivity analysis [74,85]. The values of total cutting power determined during the experimental stage were used as the target value of the cutting characteristics required in the DOE analysis to determine the parameters of the Johnson–Cook constitutive equation [30,31]. The measured distances between the neighboring chip segments formed during the short hole drilling of the studied titanium alloy were used as the target value for the cutting characteristics required in the DOE analysis to determine the critical fracture stress of the machined material according to the Cockroft and Latham model [71,79].

The multiple simulations of the cutting process envisioned by the DOE sensitivity analysis were performed using a spatial numerical model of a real machining process: the short hole drilling process. This was performed in contrast to earlier studies (see, e.g., [11,72,80]), where DOE sensitivity analysis was performed by simulating the orthogonal cutting process to determine the submodel parameters. Given the significant simulation time of one drill revolution in the drilling process, a limited number of simulation steps, not exceeding 100–150, were used to perform the DOE analysis. These simulations were performed in the steady-state region of the chip-forming process. In the first and subsequent DOE iterations, 30 simulations were performed for each set of cutting modes. In the first iteration of the DOE sensitivity analysis, broad limits of variation for each parameter of the above equation were chosen to determine the parameters of the Johnson–Cook constitutive equation. The choice of parameter variation limits was based on data from the literature (see, e.g., [39,40]) and previously performed studies [11,80]. These parameter variation limits are summarized in Table 5 (see the first iteration).

**Figure 10:**
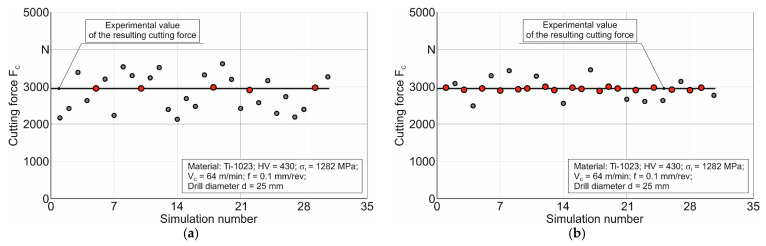
Results of the DOE sensitivity analysis for determining the constitutive equation parameters: (**a**) results of the first iteration of the DOE analysis; (**b**) results of the second iteration of the DOE analysis.

The results of the first iteration of the DOE analysis for one of the cutting mode sets are shown schematically in Figure 10a. Analyzing this figure, it can be concluded that, for the selected range of parameter variation, there are quite a few sets of constitutive equation parameters that ensure the matching of simulated cutting power values with the target value. Based on this, the limits of parameter variation were refined (see Table 5, second iteration). Schematically presented in Figure 10b, the results of the second iteration show a satisfactory number of simulations, in which the sets of required parameters are determined, ensuring the simulated cutting power values match the target value. In this way, the parameters of the constitutive equation were determined for each set of cutting conditions.

The set of constitutive equation parameters determined from the second iteration of the DOE analysis was used to determine the generalized parameter values. The generalized values of these parameters were determined by finding the intersection of the sets of similarly named parameters identified in the second iteration of the DOE analysis. This was performed according to the methodology presented in a previous study [85]. The summarized values of the required parameters are presented in Table 6. These parameter values were used in further simulations of the thermo-mechanical characteristics of the short hole drilling process in the Ti-1023 titanium alloy. The coefficients of the friction model (model of contact interaction between the drill-cutting inserts and the machined material) in different cutting zones, along with their areas, are also summarized in Table 6. The above coefficients were specified in a numerical drilling model using friction windows [84]. The values of the friction coefficients are generalized for the studied cutting modes, as well as for the outer and inner cutting inserts, following the methodology presented in earlier studies [84,85].

**Figure 11:**
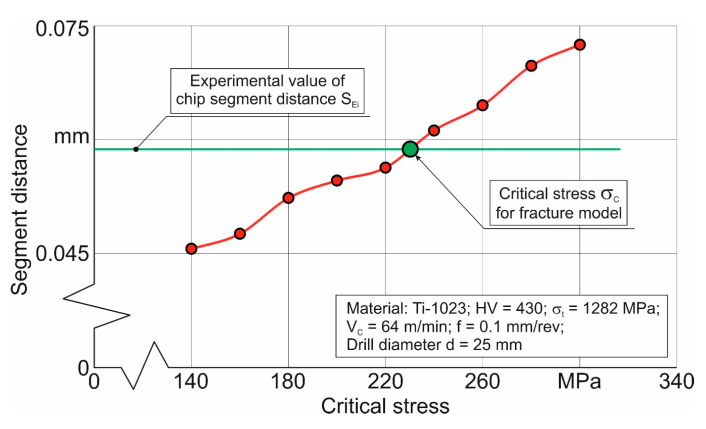
Effect of the critical damage stress in the machined material on the distance between neighboring chip segments.

Determination of the Cockroft and Latham fracture model parameter [79]—the critical fracture stress of the machined material at the penetration of cutting inserts into it—was performed by analyzing the sensitivity of the specified critical stress to the distance between separate chip segments. For example, at a cutting speed of 64 m/min and a drill feed of 0.1 mm/rev, this effect is shown in Figure 11. The measured distance between separate segments of the chip, generated at the selected cutting speed and drill feed, was used as the target value to determine the corresponding critical stress. The generalized value of the critical stress *σ_C&L_* for the studied cutting modes and for the outer and inner cutting inserts is given in Table 6. The parameters of the triad component submodels specified in this table were used in subsequent simulations of the short hole drilling process in the Ti-1023 titanium alloy.

**Figure 12:**
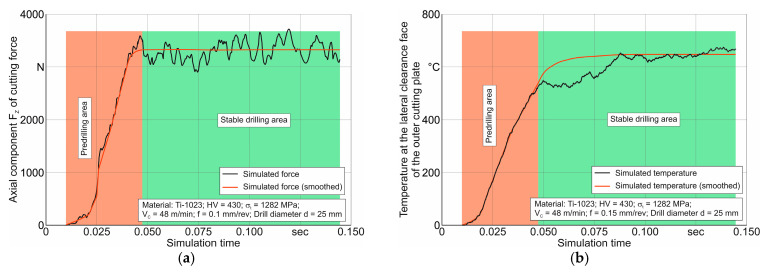
Variation of drilling process characteristics over simulation time: (**a**) variation in the cutting force axial component; (**b**) temperature change on the lateral rear face of the outer insert.

Further simulations of the thermo-mechanical characteristics of the short hole drilling process were aimed at evaluating the performance and adequacy of the drilling finite element model, using the previously determined parameters of the triad component submodels, which are suitable for simulating the cutting process of the titanium alloy Ti-1023. Initially, the results of the simulation, with signal changes in the kinetic and thermal characteristics of the drilling process, as well as the formation of chip macromorphology and other characteristics over simulation time, were analyzed. The variation in the kinetic and thermal characterization of drilling during simulation, exemplified for certain drilling process modes, is shown in Figure 12. Simultaneously with the simulated signal of the mentioned characteristics, the figure also shows the smoothed curves of the change in these characteristics over simulation time.

**Figure 13:**
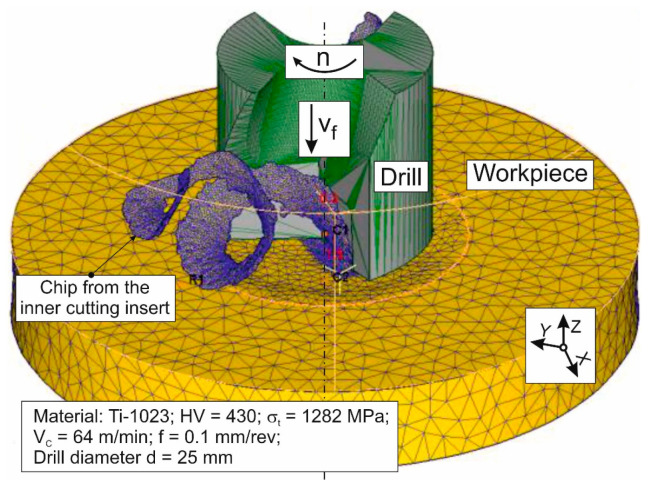
A simulation example of chip formation during drilling using an inner cutting insert.

Figure 13 shows a simulation example of chip formation generated by the inner cutting insert at a cutting speed of 64 m/min and a drill feed of 0.1 mm/rev. The generated chip has a spiral shape, which closely resembles the shape of the real chip (see Table 3) formed during the initial machining period (see Figure 6).

**Figure 14:**
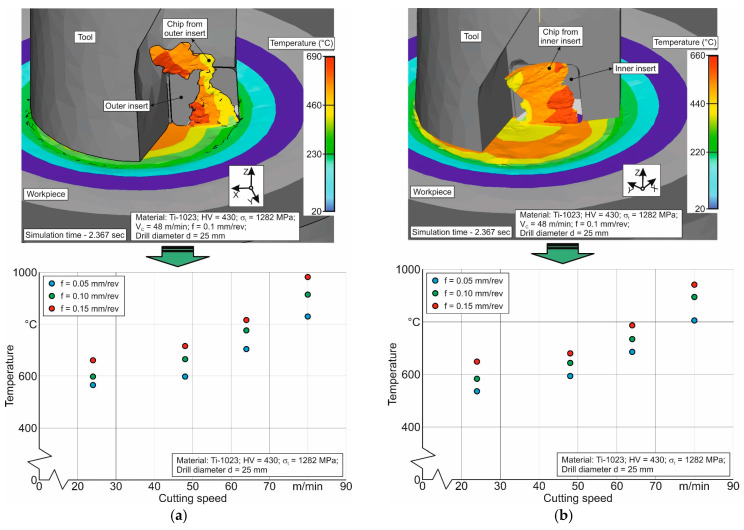
Effect of cutting modes on the temperature of chips generated by the drill’s outer and inner cutting inserts: (**a**) for the outer cutting insert; (**b**) for the inner cutting insert.

The change in the maximum chip temperature generated by the outer and inner cutting inserts, with increasing cutting speed and drill feed, is shown in Figure 14. The simulated chip temperature generated by the inner cutting insert is slightly lower than that generated by the outer cutting insert of the drill. This seems quite logical, as the average cutting speed of the inner cutting insert is less than that of the outer cutting insert [72,84].

The absolute value of the maximum chip temperature is consistent with the information presented in published sources on the cutting processes of titanium alloys [26,28,99]. Based on the simulation examples mentioned above of the short hole drilling process in the studied titanium alloy, it can be concluded that the developed numerical model is effective and can provide fairly accurate modeling of the thermo-mechanical characteristics.

**Figure 15:**
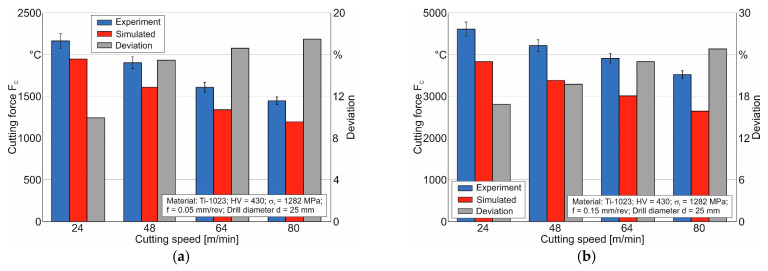
Comparison of the simulated and measured values of the resulting cutting force at different cutting speeds: (**a**) at a drill feed of 0.05 mm/rev; (**b**) at a drill feed of 0.15 mm/rev.

The final performance evaluation of the developed numerical drilling model was carried out by comparing the simulated values of the resulting cutting force and temperature on the lateral clearance face of the drill’s outer cutting insert with the corresponding measured values. Figure 15 shows a comparison of the simulated and measured values of the resultant cutting force at different cutting speeds and drill feeds.

The largest deviation between the simulated and measured values at a drill feed of 0.05 mm/rev is about 18%, while at a drill feed of 0.15 mm/rev, the deviation does not exceed 25%. In this case, the value of the deviation increases as the cutting speed increases. This indicates that the influence of the speed factor on the hardening of the machined material and its effect on the isothermal softening process with increasing speed is not sufficiently accounted for in the machined material model. This is also insufficiently accounted for in the contact interaction model (friction model) of the tool with the machined material. It should be noted that this disadvantage is realized to a lesser extent, as the friction model still has significantly less influence on the simulated values of the resulting cutting force.

**Figure 16:**
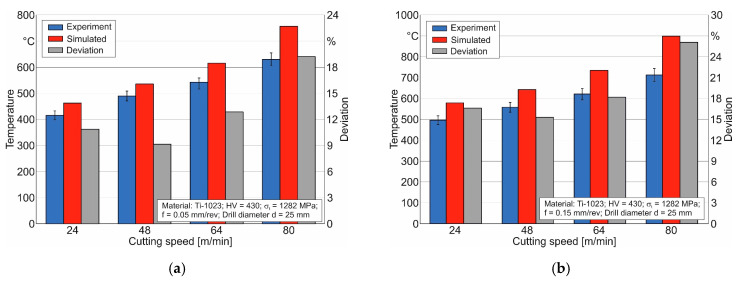
Comparison of the simulated and measured values of the temperature on the lateral back face of the drill’s outer cutting insert at different cutting speeds: (**a**) at a drill feed of 0.05 mm/rev; (**b**) at a drill feed of 0.15 mm/rev.

The comparison of simulated and measured temperature values on the lateral clearance face of the drill’s outer cutting insert at different cutting speeds and drill feeds is shown in Figure 16.

The largest deviation between measured and simulated temperature values at a drill feed of 0.05 mm/rev is observed at a cutting speed of 80 m/min, and it is about 20%. However, at a drill feed of 0.15 mm/rev and a cutting speed of 80 m/min, this deviation is 26.1%. Similar to the previous examples, the deviation between simulated and measured temperature values increases with increasing cutting speed. This once again confirms the need to clarify the possibility of the speed factor’s influence on the formation of thermo-mechanical characteristics of the cutting process. Despite these shortcomings, the developed numerical model for drilling short holes in the titanium alloy Ti-1023, as well as the methodology for determining the parameters of the triad component submodels of this numerical model, provides the ability to model the cutting process characteristics in titanium alloys. The application of such models for spatial cutting processes will significantly reduce the costs of studying these processes by potentially reducing the number of experimental studies required.

## 4. Conclusions

The subject of the presented study is the modeling of the thermo-mechanical characteristics of the short hole drilling process in the single-phase titanium ß-alloy Ti-1023 (Ti10V2Fe3Al). The parameters of the triad component submodels of the finite element model for the drilling process—the machined material model, the contact interaction model between the tool and the machined material, and the machined material fracture model—are determined using the DOE sensitivity analysis. The developed numerical model of short hole drilling was used to perform a DOE analysis. The total drilling power, determined from the measured values of the cutting force components, was used as the target value in the DOE analysis to determine the parameters of the Johnson–Cook constitutive equation (machined material model). The target value for the DOE analysis in determining the critical stress of the Cockroft and Latham model of machined material failure was the distance between neighboring chip segments. The generalized values of the parameters for the triad component submodels are determined by finding the intersection of the parameter value sets for different cutting process conditions.

The consequence of increasing the drill feed is an increase in the cutting force components and its resultant, as well as in the total cutting power. The increase in these characteristics is caused by a rise in the material volume removed per unit of time. The decrease in the resulting cutting force and its components is a consequence of the increase in cutting speed. This decrease is caused by the prevalence of isothermal softening in the machined material over its adiabatic hardening. At the same time, an increase in cutting speed leads to a higher total cutting power. In all probability, this increase is caused by the cutting speed directly affecting the calculating of cutting power. The latter significantly exceeds the decrease in the resulting cutting force.

The main chip shapes and their changes throughout the drilling process have been established by analyzing the chips generated during the short hole drilling process. An increase in cutting speed and drill feed causes a decrease in the chip compression ratio. A similar influence of cutting modes is maintained in both the cutting process by the outer cutting insert and by the inner insert. The distance between neighboring chip segments increases with higher cutting speed and drill feed. The basis for this effect of the drill feed is the increase in the degree of plastic deformation as the feed increases, which in turn leads to an increase in the formation time of the individual chip segments.

The temperature on the lateral clearance face of the drill’s outer cutting insert increases with increasing cutting speed and drill feed. The increase in the measured temperature with increasing cutting speed is a logical and repeatedly confirmed fact, as cited in numerous publications. The increase in the measured temperature caused by an increase in the drill feed is the result of a higher degree of plastic deformation in the machined material per unit of time.

Using the generalized values of the parameters of the triad component submodels in the numerical model for the short hole drilling process in the Ti-1023 titanium alloy results in a maximum deviation of no more than 25% between the simulated values of the resulting cutting force and the corresponding measured values of this force. At the same time, the maximum deviation between the simulated and measured temperature values at the lateral clearance face of the drill’s outer cutting insert is 26.1%. These deviations between the simulated and experimentally determined cutting characteristics values indicate the potential for using such numerical models to model the thermo-mechanical characteristics of the short hole drilling process in titanium alloys. In turn, this will provide an opportunity to significantly reduce costs at the design stage and optimize various cutting processes in difficult-to-machine alloys.

## Figures and Tables

**Table 1:** Chemical composition of the titanium alloy Ti10V2Fe3Al [80,88,89].

Material	Ti	Al	V	Fe	C	N	H	O	Other
Ti10V2Fe3Al	82.86–86.8%	2.6–3.4	9.0–11%	1.6–2.2%	<0.05%	<0.05%	<0.015%	<0.13%	≤0.3%

**Table 2:** Mechanical and thermal properties of the titanium alloy Ti10V2Fe3Al and drill inserts.

Material	Strength (MPa)		Elastic Modulus (GPa)	Elongation (%)	Hard-Ness	Poisson′s Ratio	Specific Heat (J/kg·K)	Thermal Expansion (µm/m·°C)	Thermal Conductivity (W/m·K)
	Tensile	Yield							
Ti10V2Fe3Al	1282 [14,89]	1220 [14,89]	110 [14]	4–10 [14]	HV 430 [14]	0.35 [14,89]	527 [90]	9.7 [90]	7.0 [90]
Drill inserts	-	-	650 [14]	-	HRC 76 [14]	0.25 [14,89]	251 [89,90]	-	59 [89,90]

**Table 3:** Comparison of chip shapes at different cutting speeds and a drill feed of 0.1 mm/rev.

Insert	Cutting Speed, V_C_ [m/min]			
	24	48	64	80
Outer	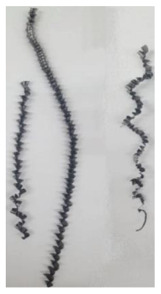	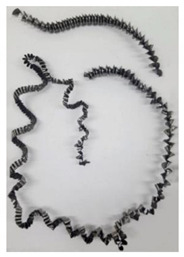	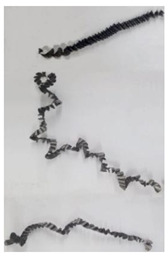	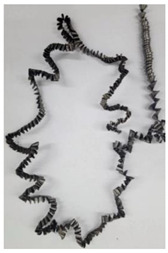
Inner	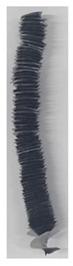	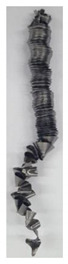	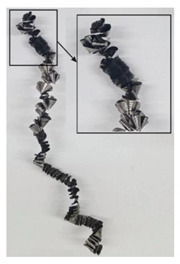	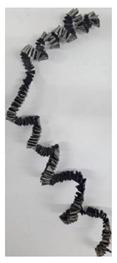
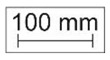				

**Table 4:** Comparison of chip microforms during a stable cutting process at different cutting speeds and a drill feed of 0.1 mm/rev.

Insert	Cutting Speed, V_C_ [m/min]			
	24	48	64	80
Outer	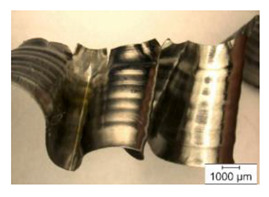	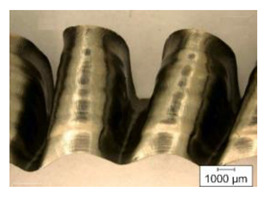	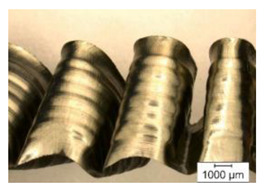	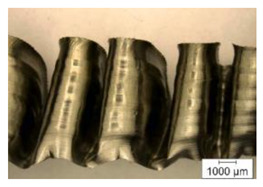
Inner	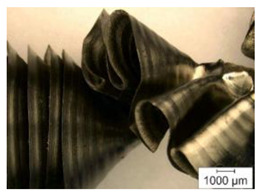	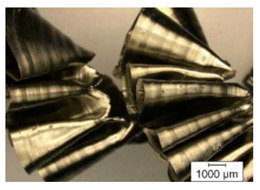	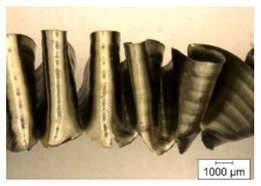	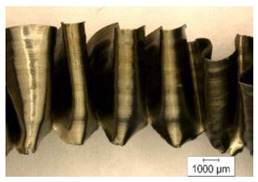

**Table 5:** Variation limits of the constitutive equation parameters for the DOE sensitivity analysis iterations.

Iteration	*A* [MPa]		*B* [MPa]		*n* [−]		*C* [−]		*m* [−]	
	Upper Limit	Lower Limit	Upper Limit	Lower Limit	Upper Limit	Lower Limit	Upper Limit	Lower Limit	Upper Limit	Lower Limit
First iteration	1400	400	1400	400	0.6	0.05	0.06	0.01	1.5	0.7
Second iteration	1100	800	900	600	0.4	0.15	0.04	0.02	1.0	0.7

**Table 6:** Parameters of the triad component submodels used in simulating the short hole drilling process.

Constitutive Equation Parameters					Friction Parameters in Cutting Zones			Fracture Stress
					Secondary Zone		Tertiary Zone	
A [MPa]	B [MPa]	n	C	m	Plastic Area, *f_RFp_*	Elastic Area, *f_RFe_*	*f_CF_*	σ_C&L_ [MPa]
945.3	745.6	0.248	0.0312	0.78	0.752	0.328	0.612	240

## Data Availability

Data are contained within the article.
